# Exploring the role of competing demands and routines during the implementation of a self-management tool for type 2 diabetes: a theory-based qualitative interview study

**DOI:** 10.1186/s12911-019-0744-9

**Published:** 2019-01-24

**Authors:** Sebastian Potthoff, Justin Presseau, Falko F. Sniehotta, Matthew Breckons, Amy Rylance, Leah Avery

**Affiliations:** 1 Faculty of Health and Life Sciences, Northumbria University, Newcastle Upon Tyne, NE7 7TR UK; 2 Institute of Health and Society, Newcastle University, Newcastle Upon Tyne, NE2 4AX UK; 3 Clinical Epidemiology Program, Ottawa Hospital Research Institute, 501 Smyth Road, Ottawa, K1H 8L6 Canada; 4 School of Epidemiology and Public Health, University of Ottawa, 600 Peter Morand Crescent, Ottawa, K1G 5Z3 Canada; 5 School of Psychology, University of Ottawa, 136 Jean-Jacques Lussier, Ottawa, K1N 6N5 Canada; 6 NIHR Policy Research Unit Behavioural Science, Newcastle University, The Baddiley-Clark Building, Richardson Road, Newcastle Upon Tyne, NE2 4AX UK; 7 Diabetes UK, London, UK; 8 School of Health & Social Care, Teesside University, Middlesbrough, TS1 3BA UK

**Keywords:** Habit, Automaticity, Routines, Intention, Dual process, Healthcare professionals, Primary care, Secondary care, Type 2 diabetes, Information prescription

## Abstract

**Background:**

The implementation of new medical interventions into routine care involves healthcare professionals adopting new clinical behaviours and changing existing ones. Whilst theory-based approaches can help understand healthcare professionals’ behaviours, such approaches often focus on a single behaviour and conceptualise its performance in terms of an underlying reflective process. Such approaches fail to consider the impact of non-reflective influences (e.g. habit and automaticity) and how the myriad of competing demands for their time may influence uptake. The current study aimed to apply a dual process theoretical approach to account for reflective and automatic determinants of healthcare professional behaviour while integrating a multiple behaviour approach to understanding the implementation and use of a new self-management tool by healthcare professionals in the context of diabetes care.

**Methods:**

Following Diabetes UK’s national release of the ‘Information Prescription’ (DUK IP; a self-management tool targeting the management of cholesterol, blood pressure and HbA1c) in January 2015, we conducted semi-structured interviews with 13 healthcare professionals (general practitioners and nurses) who had started to use the DUK IP during consultations to provide self-management advice to people with type 2 diabetes. A theory-based topic guide included pre-specified constructs from a previously developed logic model. We elicited healthcare professionals’ views on reflective processes (outcome expectations, self-efficacy, intention, action and coping planning), automatic processes (habit), and multiple behaviour processes (goal priority, goal conflict and goal facilitation). All interviews were audio recorded and transcribed verbatim and all transcripts were independently double coded and analysed using content analysis.

**Results:**

The majority of healthcare professionals interviewed reported strong intentions to use the DUK IP and having formed a habit of using them after a minimum of one month continuous use. Pop-up cues in the electronic patient records were perceived to facilitate the use of the tool. Factors that conflicted with the use of the DUK IP included existing pathways of providing self-management advice.

**Conclusion:**

Data suggests that constructs from dual process and multiple behaviour approaches are useful to provide supplemental understanding of the implementation of new self-management tools such as the DUK IP and may help to advance behavioural approaches to implementation science.

**Electronic supplementary material:**

The online version of this article (10.1186/s12911-019-0744-9) contains supplementary material, which is available to authorized users.

## Background

Translating research evidence into routine practice to improve care can be difficult and there is a wealth of research demonstrating gaps in the quality of care provided to patients [1]. A US study that included almost seven thousand patients found that less than 60% of patients received care in line with best practice guidelines [2]. Implementation science is concerned with promoting the integration of research findings and evidence into healthcare policy and practice [3] by understanding the range of factors that can prevent or enable improvements in healthcare practices [4]. A better understanding of such factors and their interactions across a range of healthcare practices has the potential for informing the design of effective implementation interventions [4]. Theories of behaviour can provide a useful lens through which implementation can be understood by describing relationships between factors that influence practice, many of which have been tested successfully in both patient [5] and healthcare professional populations [6, 7].

### Theory-based determinants of healthcare professional behaviour

Predominant behavioural approaches in implementation science view healthcare professionals’ behaviour as the result of a reflective decision-making process [7]. For example, the Theory of Planned Behaviour (TPB; [8]) suggests that the strength of a person’s *intention* (or motivation) is viewed as the most important determinant of behaviour. Two important predictors (amongst others) in Social Cognitive Theory (SCT) are *outcome expectancies* (similar to *attitudes* in the TPB) and *self-efficacy* [9]. *Outcome expectancies* refer to a person’s estimation of what the anticipated consequences of a given behaviour are [9]. *Self-efficacy* refers to a person’s perceived capability to perform a behaviour in the face of anticipated barriers to behaviour [9]. The consistent finding that intention does not always translate into action (i.e., *intention-behaviour gap*) [10, 11] has led to the development of theories that are specifically concerned with volitional cognitions such as *action planning* and *coping planning* [Health Action Process Approach [HAPA]; [12]. *Action plans* are specific plans of when, where and how to perform a behaviour and *coping plans* deal with anticipated barriers to the behaviour [13, 14]. Social cognitive and volitional models of behaviour have successfully guided both the design and evaluation of effective interventions [15].

### Habit and healthcare professional behaviour

While social cognition and volitional models provide useful insights into how behaviour is initiated, they do not sufficiently account for the role that implicit processes such as habit play in determining healthcare professionals’ behaviour. *Habit* can be defined as a learned tendency to perform a behaviour automatically in response to a specific cue in the situational context [16]. For example, the sight of a soap dispenser in a clinical setting (contextual cue) may prompt a healthcare professional to engage in hand washing without the need for explicit decision-making every time (automatic response). Taking into account that much of healthcare professionals’ behaviour might be contingent to cues (e.g. electronic reminders to prompt clinical actions) there has been a call for greater consideration of habit in behavioural theories used in implementation science [17, 18].

The suggestion that healthcare professionals’ behaviour is driven by both reflective (e.g. intention) and impulsive (e.g. habit) processes is consistent with dual process models [19, 20]. According to these models there are two internal processes that operate in parallel that determine behaviour—a reflective and an impulsive process [21]. The reflective process involves slow and effortful decision-making that operates under full conscious awareness [21]. This process is consistent with most contemporary theories of behaviour that consider outcome expectations, self-efficacy, intention and planning and there is considerable research suggesting the importance of reflection [7]. The impulsive process involves quick and efficient processes that operate outside a person’s awareness [21]. This impulsive process includes automatic action tendencies, i.e. the degree of automaticity with which the behaviour is performed. A systematic review and meta-analysis identified 9 studies assessing the strength of association between habit and healthcare professional behaviour [22]. A combined mean *r*_+_ of 0.35 was observed between habit and healthcare professional behaviour, demonstrating the impact of implicit processes on clinical behaviour. For example, a study involving 427 primary healthcare professionals (general practitioners [GPs] and nurses) tested whether a dual process model could predict the utilisation of six underperformed prescribing, advising and examining practices in diabetes care [23]. This study found that measures of both reflective and impulsive processes at baseline predicted healthcare professionals’ provision of prescribing, advising, and examining behaviours at 12 months follow-up [23].

Although quantitative evidence demonstrates the importance of habit as a predictor of healthcare professional behaviour [23, 24], there is a lack of theory-based qualitative research into the role of habit development in healthcare professionals. Qualitative research can help to triangulate findings obtained using quantitative methods (e.g., questionnaires) [25, 26] and can help to better understand how healthcare professionals form a new habit (and break old habits) and how habit subsequently impacts on behaviour. One qualitative study that took a theory-based approach and incorporated questions on habit/routines investigated barriers and facilitators to hand hygiene of healthcare professionals [27]. The study showed that habit/routine (i.e., an automatic response to cues) facilitated healthcare professional hand hygiene behaviour.

### The impact of competing demands on healthcare professional behaviour

In addition to calls for considering dual process approaches, there have also been calls for considering the role of competing demands as a way of operationalising time-related barriers [28]. Research on competing demands acknowledges the impact of *conflicting goals* and *priorities* on the pursuit of new behaviours [28, 29]. Healthcare professionals often pursue multiple goals (e.g., prescribing medication whilst maintaining a rapport with the patient), however the pursuit of one specific goal may interfere with pursuing another, for example, by taking up time available or due to incompatibility (e.g., taking blood pressure readings whilst examining a patients’ feet). The pursuit of one goal may also act to facilitate the pursuit of another, for example instrumentally (e.g., providing advice on diet can lead to setting goals for weight loss). There is quantitative and [28] qualitative research evidence [30] demonstrating the importance of going beyond single-behaviour approaches by acknowledging the impact of multiple goal pursuit. A qualitative study utilising theory-based semi-structured interviews reported that healthcare professionals readily related their other goal-directed behaviours with having a facilitating and interfering influence on two evidence-based clinical behaviours (i.e., providing physical activity advice and prescribing to reduce blood pressure) [30]. A better theoretical understanding of how competing demands influence healthcare professionals’ behaviours may provide a more representative account of the realities of clinical practice.

### Self-management support for people with type 2 diabetes

One area which may benefit from theory-based implementation work is Type 2 diabetes care. Type 2 diabetes is a worldwide epidemic affecting over 400 million adults [31]. The number of diagnosed cases in the UK has more than doubled from 1.4 million in 1996 to 3.5 million in 2015 [32]. The recognition that poor management of type 2 diabetes can lead to serious complications (e.g. cardiovascular disease, morbidity, and accelerated mortality) has led to the development of effective interventions that can halt progression and even reverse the condition [33] through health behaviour change [34, 35]. Furthermore, a large systematic review reported that self-management training in type 2 diabetes has positive effects on a range of health outcomes such as sustained glycemic control, cardiovascular disease, and quality of life [36]. As a result of this evidence, an update in clinical practice guidelines and quality standards (National Institute for Health and Care Excellence; NICE) has called for more support with self-management behaviours in patient populations [37]. To support the successful implementation of NICE guidelines healthcare professionals may require support to provide self-management advice and an evidence-informed resource could help them to deliver this evidence-based care.

### Diabetes UK information prescriptions to support self-management advice

The Diabetes UK Information Prescription (DUK IP) is a clinical tool developed to help healthcare professionals and people with type 2 diabetes to make decisions together about treatment and self-management. In the first instance, DUK released three different IPs covering three important diabetes-related health targets: blood pressure, cholesterol, and HbA1c. This intervention draws upon evidence-based behavioural science to provide a mode of targeting risk perception and supporting goal setting, action planning and coping planning of people with type 2 diabetes [38–40]. DUK IPs start with a short section including information about the three health targets (i.e., HbA1c, cholesterol, and blood pressure; [41]). This section is followed by a checkbox list of health behaviours that can be adopted (e.g., reducing portion size). An ‘agreed action plan’ section at the bottom of the DUK IP allows healthcare professionals and people with diabetes to further personalise the chosen health behaviors by formulating ‘when, where and how’ the behavior is to be adopted. DUK IPs can be used through all major primary care IT systems in the UK (i.e., EMIS Web, Vision, and SystmOne). Their installation on interconnected IT systems allows for continuous updating of the DUK IPs in the light of emerging research evidence. As Learning Health Systems (LHS) increasingly incorporate intelligent IT systems, DUK IPs have the potential to have a role within integrated online decision support and dashboard systems to support diabetes care [42, 43]. There are already several studies that show how LHS can be integrated in primary care to support the linking of routine healthcare systems with translational research [44]. For example, the TRANSFoRm EU FP7 project includes Diabetes “use cases” to enable widespread queries to identify eligible patients and use data from various federated databases [44]. Once installed on the primary care practice computers they are automatically populated with test results of people with type 2 diabetes. Completed IPs can be printed by healthcare professionals and given to the person with diabetes.

### Research questions

The DUK IPs went live in a subset of primary and secondary care practices in 2014 and healthcare professionals started to pilot them with people with type 2 diabetes. The current study aimed to capture and understand healthcare professionals’ experiences with the new tool in terms of reflective, impulsive and multiple goal processes. The following research questions were investigated: 1) How motivated were healthcare professionals to use the DUK IP? 2a) How long did healthcare professionals perceive it to take to form a habit to use the DUK IP? 2b) What contextual cues and prompts were healthcare professionals aware of that preceded their use of the DUK IP? and; 3) What other clinical activities (e.g. provision of information materials) competed with or facilitated the use of the new tool?

## Methods

### Sampling and recruitment

We aimed to recruit a purposive sample of GPs and nurses with varying years of clinical experience who had used the DUK IP. Participating healthcare professionals were recruited from primary and secondary care practices throughout the UK. Our target sample size was a minimum of 13 participants or until data saturation was reached, in line with published guidance [45]. Participating healthcare professionals included both those who were involved in the development and piloting of the DUK IP and those who had no involvement in the developmental process. The research protocol was approved by the Newcastle University Faculty of Medical Sciences Ethics Committee (Application No: 00849) and research assurance was provided by North of England Commissioning Support Unit.

### Data collection

**Fig. 1:**
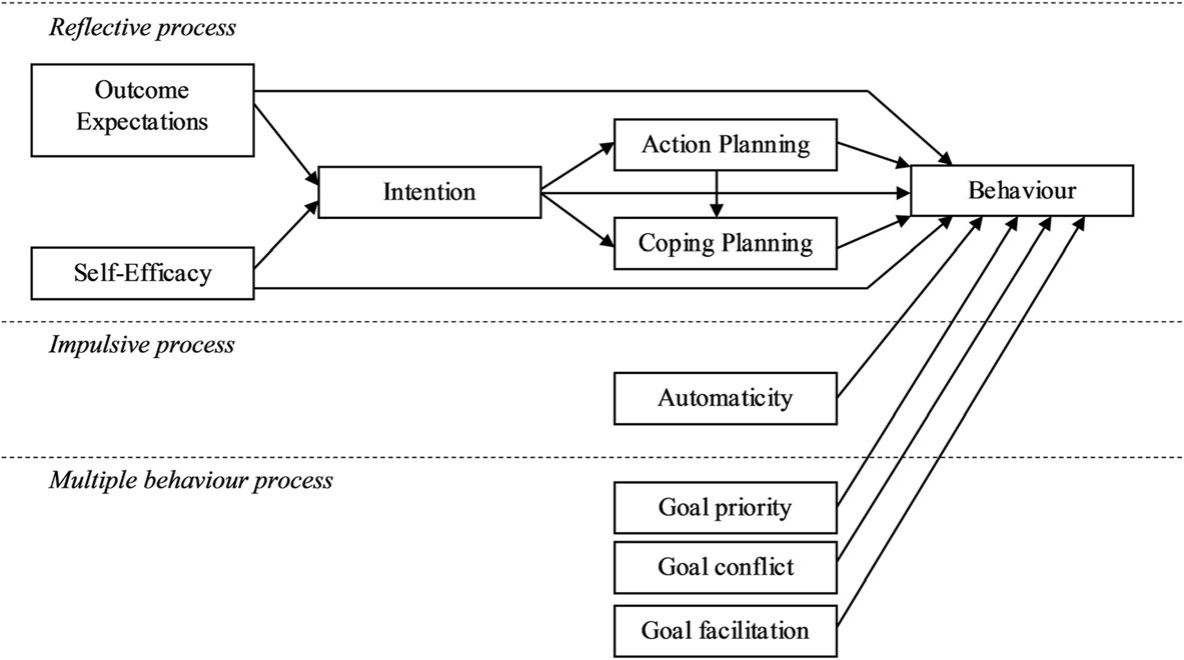
Process model of the topic guide used to facilitate interviews. The reflective process illustrates the sequential relationship between motivational (outcome expectations, self-efficacy, intention) and volitional (action planning, coping planning) factors and healthcare professional behaviour. The impulsive process shows the parallel influence of automaticity on behaviour. The multiple behaviour process acknowledges that the enactment of clinical behaviour is also influenced by the range of competing goals that healthcare professionals face in their clinical practice

Theory-based semi-structured interviews were conducted face to face or by telephone using a theory-informed topic guide (see additional file 1). This was based on a logic model (see Fig. 1) developed from a previous predictive study with healthcare professionals who were providing care to people with type 2 diabetes [23]. The topic guide included pre-specified prompts to elicit information on specific theoretical constructs included in the logic model. Specifically, we elicited healthcare professionals’ views on *outcome expectations, self-efficacy, intention, action* and *coping planning, habit, goal priority, goal conflict and goal facilitation*. The topic guide was piloted with three public health researchers at Newcastle University and with one GP prior to use in the study. The pilot indicated that interviews would take approximately 20 min. After obtaining informed signed consent from participating healthcare professionals, interviews were digitally recorded and transcribed verbatim. All interviews were conducted by SP from 5th March to the 11th November 2014.

### Analysis

A coding manual for use with NVivo 10 [46] was created, including definitions and coding instructions (see additional file 2) to ensure researchers involved in the analysis process coded transcripts consistently. Directed content analysis [47] was used to analyse interview transcripts. The predefined theoretical determinants from the topic guide were used as a guide for initial coding of the qualitative data generated, and further sub-themes were created by coders. Two coders first independently coded two transcripts then met to develop the coding manual. Then coders independently coded the remainder of the transcripts and met to resolve discrepancies. Bootstrapped estimates of Krippendorff’s alpha were calculated for each transcript to determine inter-rater reliability across all coded constructs [5000 bootstrapped samples; 48].

## Results

### Participants

A total of 14 healthcare professionals from 13 different practices across the UK were recruited. Eight (5 GPs, 3 nurses, and 1 consultant diabetologist) were directly involved in the development and/or the piloting of the new tool during its initial roll-out and the remaining five were independent of this developmental and piloting process (1 pharmacist prescriber, 1 GP, and 3 nurses). One interview was lost due to file corruption of the audio recording prior to transcription. Healthcare professionals reported a median of 18 years clinical experience (range 8–35 years) of working with patients in primary and secondary care. They had been using the DUK IP for a median of 6 months (range 2–12) prior to being interviewed.

### Interrater reliability

Krippendorff’s alpha across all constructs ranged from .52 to .88 with most alphas exceeding acceptable cut-off levels of .67 [48] indicating satisfactory agreement between coders. Illustrative quotes are provided below alongside a description of the themes and an overview of quotes for all themes is presented in additional file 3.

### Reflective process

#### Diabetes UK information prescription use (behaviour)

There was considerable variability in healthcare professionals’ self-reported frequency of using the new tool with people with diabetes that they had consulted during the week prior to the interview.

‘*I would say I print it off a couple of times a week* [2 out of 20 patients]’ (ID8);

‘*Oh, roughly I would say probably 20 a week probably* [20 out of 40 patients]’(ID13);

‘*They all get one, for Type 2* [10 out of 10 patients].’ (ID5).

#### Outcome expectancies

*Improved interaction*. The majority of the healthcare professionals observed that using the new tool helped them to improve their interaction with their patients. Healthcare professionals described how the new tool helped them to structure their consultation:

*‘It gives me an introduction, an opening conversation I can have with the patient. It’s something it can keep a consultation structured but it also allows the patients to think about things.’ (ID3).*

*Helps patient*. Respondents reported that they thought the tool helped their patients to feel more empowered and in control of their condition:

*‘So that empowers them to know a bit more about their condition and what they’re aiming for rather than just taking tablets.’ (ID7).*

They reported that the info-graphs depicting what it meant to have high blood pressure, cholesterol or blood sugar, helped raise their patients’ risk perception and thereby prompted behaviour change:

*‘I think a picture speaks a thousand words. So that is very helpful for people to see why they should do a behavioural change, because they can actually see the blood vessel getting furred up.’ (ID12).*

They also described how they believed the new tool would help their patients to better understand their condition and thereby increase their confidence to self-manage:

*‘So it means they’re able to go home and compare their figures on this to the previous one, and I think that can give them the confidence to say yes, I am doing right, I am getting there.’ (ID14).*

They further reported that the new tool prompted patients to form effective action plans that would help them to reach their behavioural/clinical goals:

*‘It clarifies everything to them so they understand what’s their goals, where they are currently and where we want them to get to, and it just clarifies the actions they’re going to be taking.’ (ID13).*

Lastly, healthcare professionals reported that they thought the agreed targets for behaviour change and for reaching the clinical goals would act as a reminder for the patient:

*‘It is an aide-memoire for the person with diabetes.’ (ID4).*

#### Self-efficacy

*Barriers affecting self-efficacy.* Healthcare professionals reported the following patient-related barriers to the use of the new tool: comorbidity (e.g., heart disease, rheumatoid arthritis, and knee pain), illiteracy, dexterity, visual problems, dementia, and lack of engagement:

*‘We have a lot of patients who have comorbidity so they’re not just diabetic but they also have heart disease and rheumatoid arthritis or whatever, so all of those things need sorting out so you might decide that actually there’s too much to do in one go.’ (ID2).*

Contextual barriers reported included lack of time and difficulties with the installation of the information prescriptions on practice computers:

*‘I think that was the biggest barrier was the installation, because I’m fairly good at IT, I’ve devised an audit tool for CKD [chronic kidney disease] and Diabetes which I’ve had published and stuff, so I’m not too bad on EMIS web, but I did really struggle just to get this.’ (ID9).*

Healthcare professionals reported low levels of self-efficacy when it came to dealing with IT-related problems and often had to seek advice to get the new tool installed on to the computer system:

*‘And, I think, if it wasn’t for the fact that we have somebody fairly specific administration team that helps with IT I might have given up at that point.’ (ID9).*

#### Intention

*Most motivated.* All but one healthcare professional were motivated to use the information prescriptions in their practice with patients with diabetes.

*‘At the moment, very [motivated], because it’s a relatively new tool, and I think they’re good’ (ID8).*

*Least motivated.* One healthcare professional reported low intentions to use the new tool, due to other competing practices that they felt were already working well:

*‘I’m probably not as motivated as others because of the tools I’ve already devised myself’ (ID9).*

They reported a range of situations in which they were least motivated to use the new tool, including patient-related situations such as when the nature of a consultation made the provision of an IP inappropriate:

*‘If a patient has come in, the consultation, if it has been around a particularly sensitive topic or emotional topic, a bereavement it wouldn’t be appropriate to be talking about control of their diabetes at that stage’ (ID4).*

Context-related situations were also described including those in which patients were perceived to have practical difficulties using IPs:

*‘One patient I gave it to her and she said “I really don’t know how to decipher this. I lost one of my children”. But she’s not come back so I think people who English is not their first language or they find it difficult to read, they will have difficulty in engaging with this.’ (ID2).*

#### Action planning

A minority of healthcare professionals reported having a clear plan for when, where and how they would use the new tool with their patients. A patient requesting further diabetes-related information was an opportunity during which healthcare professionals used the tool:

*‘When the patients come in and they ask can you tell me what my latest diabetes control blood test was like, that’s when I’d then bring in that one [information prescription]’ (ID11).*

A further opportunity for healthcare professionals to use the new tool was related to the time in the consultation, with the end of the consultation being a preferred time:

*‘And it is at the end bits gathering all the information, this is where we think you are, and have a look at this, what do you think you can do to help’ (ID8).*

#### Coping planning

Healthcare professionals sought help from relatives and translators in situations where their patients were unable to understand the information presented on the IPs:

*‘I have an interpreter that works with me in my community clinic, and some family members come but I’ve always got an interpreter’ (ID11).*

They also made use of the info-graphs to explain the information to non-native speakers:

*‘A lot of my patients are from different countries so English is not their first language, so I find that this is, the picture, is very easy for them to understand’ (ID3).*

In situations where healthcare professionals encountered contextual barriers (i.e. lack of time) they either deferred use of the new tool to a later time or they asked a diabetes specialist nurse to discuss the content with the patient:

*‘You park that and say let’s do that another day or come and see the nurse another day and do that with her.’ (ID12).*

### Impulsive process

#### Contextual cues

All except one healthcare professional reported that they had access to the electronic pop-up reminders that appeared in patients’ electronic records when one of the three targets (i.e. blood pressure, cholesterol or glycemic control) was outside the recommended range:

*‘There’s a little pop-up screen at the right-hand corner, and that says diabetes information prescription, so that’s a memoire for you’ (ID6).*

The majority of the responses of healthcare professionals indicated that if installed appropriately the pop-up reminders promoted their use of the new tool:

*‘So that was the single most useful thing [pop-up reminder], and that’s how I first became aware of them, and that’s why I keep remembering about them’ (ID10).*

Healthcare professionals also reported that people with diabetes (i.e. patients) acted as a social prompt to provide the new tool:

*‘Some patients are actually asking for them. Can I have the paper we had last time and what can we do this time’ (ID14).*

#### Habit formation

The vast majority of those interviewed reported that they used the new tool automatically, without having to think about it consciously:

*‘Because I’ve been using it for so long [12 months] it has become a sort of subconscious way of using it rather than I have to remember to do it. You normally do it and it just happens’ (ID6).*

They reported that it took them between one and three months of repeated use to use the new tool on a routine basis:

*‘I think it’s the old adage that you use something for a month it gets into a habit. It’s become a habit now’ (ID14).*

*‘It probably took about a couple of months to get into the actual habit of it but now it’s a routine thing that during the consultation it’s printed off’ (ID4).*

### Multiple goal process

#### Goal priority and goal conflict

Healthcare professionals reported a range of goals that took priority over the use of the information prescriptions. Treating comorbidities that occurred alongside diabetic symptoms were perceived as having higher priority:

*‘We have a lot of patients who have comorbidity so they’re not just diabetic but they also have heart disease and rheumatoid arthritis or whatever, so all of those things need sorting out so you might decide that actually there’s too much to do in one go.’ (ID2).*

They also reported prioritising their goals according to the needs of their patients:

*‘I would go first of all according with the patient’s reason for coming along and then I will say just looking at your notes before you came in I can see that we could be doing a little bit more for you and that’s how I’d introduce it.’ (ID4).*

Furthermore, healthcare professionals reported other administrative tasks often taking priority over the use of the information prescriptions:

*‘If you’ve got about 4 different forms to fill like dementia and unplanned admissions and you’ve got a bit of QOF [Quality Outcome Framework] to do then this would take a little bit of lesser priority’ (ID6).*

A minority of healthcare professionals reported that they had been using alternative self-management resources and strategies. For some the new tool had substituted previously used self-management resources and strategies, whereas others kept on using competing methods, which conflicted with their use of the DUK IP:

*‘We did have our own care plans, […]. And that was all on one piece of paper, and then we had a little action plan that we wrote out for them. So when these ones [information prescriptions] came in I had probably not used them as extensively as maybe other surgeries would because we had already got our own care plan that we were using.’ (ID9).*

## Discussion

This qualitative interview study applied a dual process model of healthcare professional behaviour supplemented by a multiple goals approach to better understand the determinants involved in the implementation of a new self-management tool, the Diabetes UK Information Prescription. Findings suggests that the uptake of the new tool could be explained by a combination of reflective (e.g. intention) and impulsive, non-conscious processes (e.g. cues, habit). Furthermore, we found evidence that conflicting goal-directed behaviours contributed to the extent to which healthcare professionals reported making use of the new tool.

While previous studies have applied dual process [23] and multiple goal models [30, 49] separately to investigate clinical behaviours, the current study is the first to apply both models simultaneously. Given the consistent finding that the translation of evidence-based practices into routine care can be a slow process involving healthcare professional behaviour change [50], these findings have the potential to inform the further implementation of the DUK IP and/or other interventions.

The majority of healthcare professionals in the current study reported high intentions and positive outcome expectancies regarding the use of the new tool with their patients. The finding that reflective processes, as represented in most social cognitive models of behaviour, are an important determinant of healthcare professionals’ behaviour is consistent with findings in the implementation literature [51]. For example, a literature review including 31 studies found that intention was an important determinant of healthcare professionals’ use of health information systems [52]. Although one factor that may have biased views towards a positive evaluation of the tool could have been that some of the participating healthcare professionals were directly involved in the development of the tool. This is in line with research suggesting that the active involvement of users in the implementation of new interventions can promote a sense of ownership towards the intervention [53].

The majority of healthcare professionals interviewed reported that after at least three months of continuous use they had formed a habit, or an automatic way of using the new tool. Although this is not the first study that has found evidence that habit is an important driver of healthcare professional behaviour [24, 54, 55], this is the first qualitative study to our knowledge that examined habit formation and automaticity development in the use of a new self-management tool. Healthcare professionals reported that one of the most important facilitators for their use of the DUK IP was the integrated prompts in the electronic patient records. This finding is in line with the literature supporting point of care reminders in healthcare professionals [56, 57]. For example, a systematic review including 32 studies found that computer-generated reminders had a moderate effect on improvement in healthcare practices [56]. Another systematic review of 28 studies found that computer reminders achieved a median improvement in process adherence of 4.2% [57]. From a habit perspective reminders might be particularly useful as they help to maintain a behaviour that has become habitual, and increase behavioural automaticity [58]. Taken together this evidence suggests that the use of electronic reminders may be a beneficial strategy to facilitate the use of information technologies, such as the information prescriptions.

Results showed that healthcare professionals perceived other goal directed behaviours as interfering with the use of the new tool. These results are consistent with other qualitative studies with patients [59] and healthcare professionals [30] that report the interfering effects of other goal pursuits on the performance of a focal behaviour despite strong intention. For example, some healthcare professionals were already using alternative, competing practices (e.g. alternative strategies to provide self-management advice, including information leaflets) that would directly compete with the use of the new tool. Given the limited time and resources that healthcare professionals have available during consultations, it is important to understand the range of different goals that compete for their attention.

### Implications for theory development in implementation science

The implementation process includes understanding the behaviours of frontline healthcare providers who are expected to use evidence to inform their own practice [6]. Theories of behaviour can be applied to help build a cumulative science to better understand the processes that drive healthcare professional behaviour. Most contemporary theories focus on explaining single behaviours that are assumed to be driven by a reflective decision-making process [7, 60]. The current study adds to a growing body of literature, which acknowledges that healthcare professionals’ behaviours are driven not only by a reflective process of active decision-making, but also by more impulsive processes that trigger behaviour automatically in response to contextual cues [17, 23, 24]. Furthermore, the theoretical framework that was applied in the current study did not look at behaviour (i.e. information prescription use) in isolation, but also acknowledged that new behaviours need to be integrated into a network of existing behaviours that have facilitating and interfering effects on each other.

### Implications for implementation support

The current study can provide some guidance on how to promote the implementation of new self-management tools such as the DUK IP. One way of supporting behavioural repetition (and habit formation) could be through the effective use of electronic pop-up reminders that prompt healthcare professionals on when to initiate a new behaviour [56]. However, some healthcare professionals reported problems relating to the installation of the new tool on their computer systems. This is in line with other research showing that *ease of use* is one of the most important determinants of healthcare professionals’ engagement with new technologies [61]. Future implementation interventions may need to provide additional support for the installation and use of information technologies to promote regular use.

This study also showed that effective implementation of new behaviours might need to be combined with the de-implementation of competing non-evidence based practices. For example, a minority of the healthcare professionals reported using alternative ways of providing self-management advice which might conflict with using the DUK IP. This is a challenge as research has shown that changing healthcare professionals’ behaviour is difficult particularly if it involves changing existing routines and practices [62]. It has been suggested that to break a habit one needs to overrule the impulsive system by engaging the reflective system [20]. This process can be cognitively challenging and involves inhibiting activated habit responses. Such demanding self-regulatory processes might be hard to initiate in the stressful, time constrained context of clinical practice [63]. An alternative approach could be to remove cues that trigger the old habit (e.g. non-evidenced information leaflets), making it possible for healthcare professionals to consciously consider other behavioural alternatives. This raises the importance of designing decision support tools (such as the DUK IPs) that fit within a learning health system. Such tools need to have mechanisms in place to ensure systematic decommissioning of low-value-care practices [42, 43]. For example, the DUK IPs can be kept up to date via the primary care IT system (top-down control) or de-activated locally on the practice computers (bottom-up control). Future decision-making support systems need to have similar processes in place to ensure their decommissioning when they are no longer supported by evidence or when new and better interventions come to light. If the removal of cues is not feasible, planning strategies could be used to connect old habit cues (e.g. patient asking for information) with more desired responses that are in line with the evidence on best practice [18].

### Strengths and limitations

This study used directed content analysis to test an explicit and a priori-defined theory in the context of the implementation of a new evidence-informed tool (i.e. DUK Information Prescriptions) in diabetes care. The theory-guided method helped contribute to a cumulative science that aims to understand the factors (e.g. intention and habit) that may drive healthcare professional behaviour. A limitation is that we only included healthcare professionals who were already using the DUK IP. The study could have benefited from inclusion of healthcare professionals who were not yet using the DUK IP. We focused on active users of the DUK IP as we were specifically interested in the process of habit formation and how the use of the DUK IPs fit in with healthcare professionals’ competing demands. Furthermore, the finding that healthcare professionals reported habit formation to take at least between one and three months of continuous use needs to be interpreted with caution as the frequency with which different healthcare professionals consulted people with type 2 diabetes may have varied considerably. For example, diabetes specialist nurses may have utilised the DUK IPs more regularly than practice nurses or GPs. Future research should consider the frequency of behavioural repetition as well as the time period when investigating habit formation. Lastly, this study is limited in that it only included one healthcare professional per practice. Future studies should include a number of healthcare professionals per site to understand how different people work together to implement the DUK IPs. An approach such as the Normalization Process Theory [NPT; 64] may help inform such explorations as it provides a number of generative mechanisms of social action. For example the construct ‘Activation’ refers to the need for people to collectively define the actions and procedures that are needed to maintain a practice and stay involved [64].

## Conclusion

Healthcare professionals perceived that both reflective (e.g. intention) and impulsive (e.g. habit) processes had an impact on their adoption of a new national ‘DUK Information Prescription’ for diabetes. Furthermore, they reported that other goal-directed behaviours such as competing practices influenced their adoption of the information prescriptions. Taken together data suggests that constructs from dual process and multiple goals approaches are useful to understand how new medical interventions are implemented into routine practice.

## Additional files

Additional file 1: Theory-informed topic guide.pdf. Pdf file. (PDF 192 kb)

Additional file 2: Coding tree based on theory-based process model.pdf. Pdf file. (PDF 194 kb)

Additional file 3: Illustrative quotations by themes.pdf. Pdf file. (PDF 83 kb)

## Abbreviations

CKD: Chronic kidney disease
DUK IP: Diabetes UK information prescription
EMIS: Egton Medical Information System
GP: general practitioner
HAPA: Health Action Process Approach
NICE: National Institute for Health and Care Excellence
NPT: Normalization Process Theory
QOF: Quality Outcome Framework
SCT: Social Cognitive Theory
TPB: Theory of Planned Behaviour
UK: United Kingdom
USA: United States of America
