# Supplementary material for: Exploring the role of competing demands and routines during the implementation of a self-management tool for type 2 diabetes: a theory-based qualitative interview study
Source: BMC Med Inform Decis Mak. 2019 Jan 24;19:23. doi: 10.1186/s12911-019-0744-9 (PMC6345053; doi:10.1186/s12911-019-0744-9)
Supplement: Supplementary file 1 — Theory-informed topic guide.pdf. Pdf file. (PDF 192 kb) [file 12911_2019_744_MOESM1_ESM.pdf]

Additional file 2: Theory-informed topic guide.pdf. Pdf file.

Introduction

1. Introduce researcher and purpose of the study
2. Obtain consent to proceed and to record the conversation
3. Remind interviewee that all information remains confidential, and that they are free to stop the interview and withdraw at any time.

Demographics

- What is your job title?
- How many years of experience do you have?
  - How many years working with people with type 2 diabetes?

Theory domain questions

Behaviour

- Since starting to use the info prescriptions, how many patients a week have you seen?
  - And of those, how many did you use the info prescription with?

Outcome expectancy

- How useful has the information prescription been for improving the interaction between you and your patients?
  - In what ways has it been useful?
- Can you talk to me a bit about how you think this tool might help your patients improve their HbAc1 levels, blood pressure, and cholesterol levels? (might be difficult)

Intention

- How motivated are you to use the information prescription?
- When are you most motivated?
  - Keep prompting for examples (e.g. when prompted by patient, when reminded by the computer, when enough time)
- When are you least motivated?
  - E.g. when stressed, when patient appears to know about self-management

Action planning

- Do you have a specific plan for when, where and how you will be using the information prescription during your consultation?
  - If so how did you plan the way you are using the information prescription?

Self-efficacy

- Did anything make it difficult or impossible to use the information prescription (barrier)?
  - If so what? (have prompts ready in case they aren't sure)
  - E.g. Discussing weight management is a sensitive topic, patient doesn't believe in lifestyle changes, the ink of the printer is running low, running low in time
- How confident are you that you would still be able to make use of the information prescription even if those barriers come up?
- Did anything make it easier to use the information prescription? If so what?
  - E.g. patient asking about self-management advise, printing them out before an appointment

Coping planning

- When you encountered any barriers (come up with some examples) did you have a plan in place on how to deal with the barriers so that you could still make use of the information prescription despite the barriers?

Automaticity

- What triggered you to use the information prescription (If not clear, ask what made you think of using it in the consultation? Something you did? Something the patient said? Prompt on the screen? Having them on the desk?). Did you only ever use it when trigger occurred or other times too?
- Do you see the use of the information prescription as part of your regular routine yet? If so, what supported that? If not, what might help it to become more routinely used?
- How long do you think it will take to build this into your routine? What would it take for you to use it without having to remember?

Competing demands

- I imagine you have been delivering this type of advice in the past in different ways. What kind of ways have you previously used/are you using to convey the same information (e.g. HbAC1 levels, high blood pressure, and high cholesterol levels)?
- How does the new information prescription compare to alternative methods? Has the information prescription replaced your other methods? If not, why?

Final section

- Do you have any other thoughts or suggestions?
- The information prescription will be rolled out and installed on all the computer systems in early autumn. Do you have any advice about how this can be done in the most helpful way?

End of Interview

Thank respondent for their time and switch off recorder.
