# Supplementary material for: Exploring the role of competing demands and routines during the implementation of a self-management tool for type 2 diabetes: a theory-based qualitative interview study
Source: BMC Med Inform Decis Mak. 2019 Jan 24;19:23. doi: 10.1186/s12911-019-0744-9 (PMC6345053; doi:10.1186/s12911-019-0744-9)
Supplement: Supplementary file 2 — Coding tree based on theory-based process model.pdf. Pdf file. (PDF 194 kb) [file 12911_2019_744_MOESM2_ESM.pdf]

Additional file 3. Coding tree based on theory-based process model.pdf. Pdf file.

a. Reflective process
    i. Behaviour
    ii. Outcome expectancy—*The HCPs estimate of whether the usage of the information prescriptions will lead to a certain outcome.*
        1. Improve interaction
        2. Improve patient outcomes
    iii. Intention—*An indication of the HCPs readiness (or motivation) to use the information prescriptions. It is assumed to be an immediate antecedent of behaviour.*
        1. Most motivated
        2. Least motivated
    iv. Action planning—*The extent to which HCPs have a specific plan when, where and how to use the information prescriptions.*
    v. Self-efficacy—*The HCPs' perceived capability to use the information prescriptions, even in the face of potential barriers*
        1. Barriers
        2. Facilitators
    vi. Coping planning—*The extent to which a HCP has a plan of how to deal with barriers to the usage of the information prescriptions.*
b. Impulsive process
    i. Automaticity—*The extent to which the usage of the information prescriptions has become a habit or routine.*
        1. Contextual cues—*Cues or prompts that remind the HCP to use the information prescriptions (e.g. pop-up, something the patient says, or having a stack of printed information prescriptions on the desk)*
        2. Habit formation
c. Multiple behaviour process
    i. Competing demands—*Alternative methods of delivering similar advice (e.g. leaflet by The British Heart Foundation) and that might compete with the usage of the information prescriptions.*
