# Supplementary material for: Exploring the role of competing demands and routines during the implementation of a self-management tool for type 2 diabetes: a theory-based qualitative interview study
Source: BMC Med Inform Decis Mak. 2019 Jan 24;19:23. doi: 10.1186/s12911-019-0744-9 (PMC6345053; doi:10.1186/s12911-019-0744-9)
Supplement: Supplementary file 3 — Illustrative quotations by themes.pdf. Pdf file. (PDF 83 kb) [file 12911_2019_744_MOESM3_ESM.pdf]

Additional file 4. Illustrative quotations by themes.pdf. Pdf file.

	**Illustrative quotation**
**Category: Reflective process**	
1. Theme: Behaviour	"I probably used it on maybe about 20 or 30 percent because of the fact that we'd still been using our other tool." (ID9)<br><br>"Not everyone because some of them are actually nicely controlled so once they're controlled they don't really need it so I would say probably about 15 percent. 15 to 20 percent at the very most." (ID4)<br><br>"They all get one, for Type 2, they all get it [information prescription]." (ID5)
2. Theme: Outcome expectancies	
2.1. Sub-theme: Improve interaction	"I think it enhances it. I always try and build good rapport anyway, because the diabetes management and for behavioural change you need that. So this is an extra string to my bow." (ID12)<br><br>"I know the patients very well and the families very well so I suppose it helps us to focus, otherwise it's very easy to go off on a tangent on something else." (ID14)
2.2. Sub-theme: Helps patient	"Well it's all about empowering our patients isn't it? So giving them the information, giving them the knowledge." (ID10)

	"I believe that a patient who is knowledgeable about their own conditions are better at dealing with their condition" (ID2)
3. Theme: Intention	
3.1. Sub-theme: Most motivated	"I suppose I'm a self-selecting population. I'm a diabetes specialist, that's one of my interests, my passion, so I will be more motivated than the average clinician to use it." (ID10)<br><br>"I've been part of the process so I'm quite highly motivated" (ID4)
3.2. Sub-theme: Least motivated	"If the patient says no, I want to talk about my knee pain only then it's heavy-handed to say no, I'm going to talk about this as well." (ID2)<br><br>"I think if I've very busy and when you're short on time then I probably won't use it on that occasion but then it will pop up the next time so it gives me a thing to do there." (ID4)
4. Theme: Action planning	"When I'm just drilling in or homing in on one particular thing then I will use them." (ID9)<br><br>"Now clinically I would always target the blood pressure first, and then I'd target the cholesterol, and then I'd target the HbA1c." (ID7)
5. Theme: Self-efficacy	
5.1. Barriers that reduce self-efficacy	"If they're not to hand or I don't have a printer or

	something that's when I'm less likely to do it because I'd have to go and look for it, so I'm less likely to use it then." (ID13)<br><br>"It would be the fact that they may, actually, also have mental health issues and that needs sorting out." (ID2)
5.2. Facilitators that enhance self-efficacy	"So they need to be printed out and stapled and given out already, so that's in an ideal situation it would already be done for me." (ID12)<br><br>"[…] because they are there on the computer. They're set up, it's one click of the mouse and it's there for you." (ID14)
6. Theme: Coping planning	"I have had patients who've said I don't want to talk about it right now, I'm in a rush. Fair enough. You park that and say let's do that another day or come and see the nurse another day and do that with her." (ID2)<br><br>"I have an interpreter that works with me in my community clinic, and some family members come but I've always got an interpreter." (ID11)
**Category: Impulsive process**	
7. Theme: Automaticity	
7.1. Sub-theme: Contextual cues	"That pop-up box is a really good reminder that if I wanted to I could offer them a care plan." (ID9)<br><br>"But the way we work through it is you can see at the

	bottom, there's a little pop-up screen at the right-hand corner, and that says diabetes information prescription, so that's a memoire for you." (ID6)
7.2. Sub-theme: Habit formation	"Once I was aware of them I got into the habit." (ID10)<br><br>"Like anything new it wasn't easy getting other team members to do it but now they're used to it they're fine." (ID3)
**Category: Multiple behaviour process**	
8. Theme: Goal priority	"The amount you get prompted and plus as a GP I have lots of conflicting priorities and it depends on the time of the year so at the moment we have to hit targets for the QOF which ends in the end of March, so those will take priority." (ID2)<br><br>"[…] the challenge is how do you fit in one extra intervention like this when you've got many of the pressure on your time." (ID6)
9. Theme: Goal conflict	"[…] the patient has of unplanned admissions and they're quite lengthy and quite chunky, quite meaty bits of work that you need to do and you've got your usual QOF which keeps reminding you x, y, z isn't addressed. (ID6)
10. Theme: Goal facilitation	"[…] you just say are we going to talk about these things, what do you think, and they might say yes, great idea, but I also want to talk about 5 other things then you might say well, actually, hang on, we'll just talk

	about 1 of those things and 1 of these things<br><br>[information prescriptions], how about that. (ID10)
